# Supplementary material for: Prognostic Impact of EGFR Amplification and Visceral Pleural Invasion in Early Stage Pulmonary Squamous Cell Carcinomas Patients after Surgical Resection of Primary Tumor
Source: Cancers (Basel). 2022 Apr 27;14(9):2174. doi: 10.3390/cancers14092174 (PMC9101408; doi:10.3390/cancers14092174)
Supplement: Supplementary file 1 [file cancers-14-02174-s001.zip › cancers-1673979-supplementary.pdf]

Supplementary Table S1. Commercial antibodies used in this study.

Antibody	Clone	Dilution	Manufacturer	Interpretation
TTF-1	SPT24	1:200	Novocastra ™ Leica Biosystems	Negative: No nuclear stain in any tumour cell. Positive: Nuclear stain.
p40	BC28	Prediluted	BOND Ready-to-Use Primary Antibody. Leica Biosystems	Negative: No nuclear stain in any tumour cell. Positive: Nuclear stain.
EGFR	EGFR.113	1:20	Novocastra ™ Leica Biosystems	(0/+): No membranous stain or low expression in tumour cells; (++): 1-35% of tumour cells with membranous stain. (+++) <35% of tumour cells with membranous stain. .

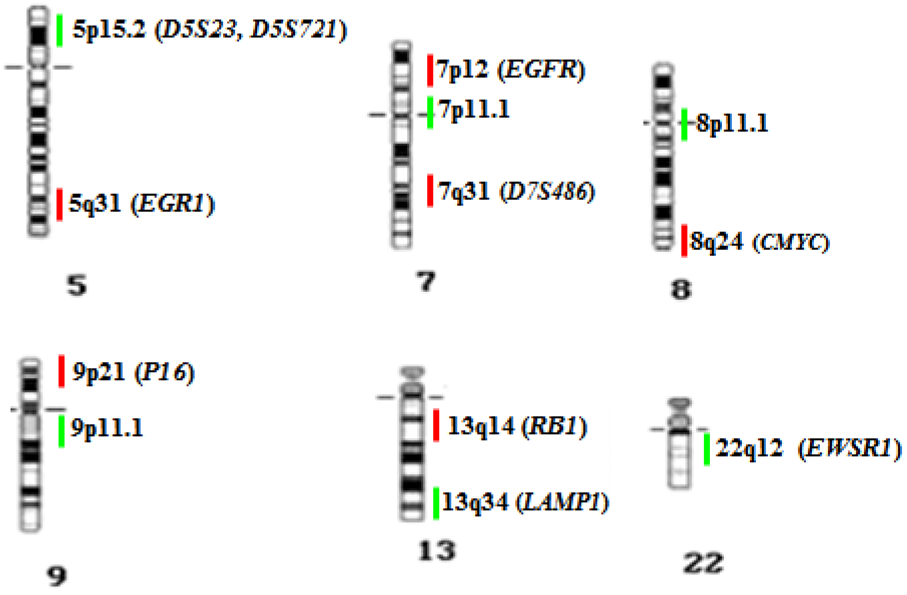

Supplementary Figure S1. Chromosome distribution of the FISH probes directed against 12 different specific genes, loci and centromeric regions of the 6 human chromosomes, used in the present study. Green and red lines correspond to the Spectrum Green and Orange fluorochromes conjugated to the probes, respectively. All probes were purchased from Vysis Inc (Chicago, IL, USA).
